# Theoretical understanding of contextual motivations for sustained adolescent marijuana use in South Africa

**DOI:** 10.4102/sajpsychiatry.v27i0.1615

**Published:** 2021-07-29

**Authors:** Emmanuel Manu, Mbuyiselo Douglas, Mohlomi J. Ntsaba

**Affiliations:** 1 Department of Population and Behavioural Sciences, School of Public Health, University of Health and Allied Sciences, Hohoe, Ghana; 2 School of Nursing and Public Health, University of KwaZulu-Natal, Durban, South Africa; 3 Department of Nursing, Faculty of Health Sciences, Walter Sisulu University, Mthatha, South Africa

**Keywords:** motivation, marijuana use, adolescence, vulnerable populations, substance abuse

## Abstract

**Background:**

Although various reasons for adolescent marijuana use have extensively been explored, contextual factors that sustain the practice in settings where the plant is illegally cultivated, especially in South Africa, remain a grey area.

**Aim:**

We aimed to explore the contextual factors of sustained adolescent marijuana use in two illicit marijuana-growing settings of the Ingquza Hill Local Municipality of South Africa, based on the differential opportunity theory (DOT) and subcultural theory (SCT).

**Setting:**

The study was conducted in two illicit marijuana-growing communities in the Ingquza Hill Local Municipality of the Eastern Cape Province of South Africa.

**Methods:**

Exploratory qualitative research, using focus group discussions approach, was conducted amongst 37 participants, four focus groups and in two communities in the Ingquza Hill Municipality of the Eastern Cape Province of South Africa. Purposive and snowball sampling techniques were used to select the communities and participants, respectively. The data were analysed using a thematic content analysis approach and presented under various themes.

**Results:**

Nine themes, grouped under two broad factors, DOT influences (availability and affordability of marijuana, idleness and means of dealing with personal problems) and SCT influences (peer conformity, the pleasure derived from marijuana smoking, manipulation of appetite, health reasons, for higher cognitive function and addiction), emerged from the analysis.

**Conclusion:**

As marijuana has been identified to be a gateway drug for the use of other illicit drugs, its sustained usage amongst adolescents poses a health challenge to the user, community and the country’s healthcare system at large. Hence, there is the need to intensify adolescent marijuana use prevention campaigns in illicit marijuana-growing contexts of South Africa, focussing on the differential opportunities and subcultural inclinations that promote the behaviour in those contexts.

## Introduction

Adolescent substance use is a major public health issue confronting many countries,^1,2^ including South Africa.^3^

Mid-to-late adolescence, also referred to as emerging adulthood, has been identified as the period when substance use is highly pronounced.^4,5^ During adolescence, contextual factors such as peer conformity, availability of substances in one’s environment and curiosity, amongst others, greatly influence substance use.^6^ Although adolescents from all walks of life are confronted with substance use, those from neighbourhoods where illicit drugs are produced are at most risk. This is often a result of the availability and ease of accessibility to drugs in such settings.^7^

Several black South African communities are either socially or economically marginalised as a result of apartheid,^8,9^ and therefore, are the case of communities in the Ingquza Hill Local Municipality in the Eastern Cape Province.^10^

The lack of socio-economic opportunities in the municipality often pushes most inhabitants into illicit marijuana cultivation and trading for survival,^11,12,13^ making the drug easily available and accessible for use.

It has been reported that in neighbourhoods where children are exposed to illicit drugs, there is often the culture of early childhood introduction to illicit marijuana usage^14^ as they often lack the willpower to make informed decisions.^15^ Although some children can stop illicit drug use during adolescence, others grow up with the behaviour.^16^ There is therefore the need to focus on understanding the factors that influence sustained adolescent drug use in settings where illicit drug production is rife, aside from the availability and accessibility of drugs to inform policy formulation.

Whilst research has been conducted on adolescent marijuana use in the South African context, these studies are often quantitatively inclined,^17,18^ without considering the context that promotes the behaviour. However, to effectively develop and implement a successful adolescent marijuana use preventive programme, especially for those in illicit marijuana-growing settings, it is prudent to understand the contextual influences of the behaviour from a theoretical perspective.^19^ Therefore, this study was framed with the differential opportunity theory (DOT) and subcultural theory (SCT), because of their dynamism in explaining the contextual influences of delinquent behaviours such as marijuana use.

Proposed by Cloward and Ohlin,^20^ DOT focusses on understanding the causes of delinquency and criminality amongst gang members. Proponents of the theory opine that using criminal and illegal means of opportunity requires a set of learned skills, just like those involved in legitimate means. Thus, people’s access to both licit and illicit means of wealth generation is socially structured. Thus, there is a ‘differential opportunity’ for reaching economic goals either by legitimate or illegitimate means. Hence, communities that face economic marginalisation and deprivation will use whatever means necessary to meet their needs and earn a living, whether legal or illegal. People invariably learn a set of skills based on where they live and the economic opportunities that their environment presents to survive.^21^

Consequently, if those sets of learned skills are geared towards illicit behaviour such as marijuana cultivation or trading, then they are likely to be influenced to use it. Aside from the set of skills that one possesses, other forms of marginalisation and disenfranchisement of communities such as unemployment, lack of economic opportunities and mobility also lead to sustaining illicit behaviours such as marijuana use, especially in environments where illicit cultivation and trading of the plant are rife.^21^ In the United States, for instance, unemployment has been found to be related to drug and alcohol use.^22^ The lack of economic opportunities in one’s environment and the inability to move out of such an environment to search for a job further worsen the unemployment situation, leading to idleness and despondency, thereby increasing the likelihood of drug use and addiction.^23^

Often, the lack of employable skills makes it difficult for people to move out of their limitations in search of jobs as they are likely not to receive any form of gainful employment. Those who take the risk to move out often get trapped in low-wage jobs because of their lack of skills and/or education,^24^ encouraging participation in illicit economic activities such as marijuana cultivation, which leads to habitual drug usage.

On the other hand, SCT suggests that deviance is the result of individuals conforming to the values and norms of a social group to which they belong.^25^ Thus, if an individual joins a social group whose norms differ from those of the main society, then such a person is likely to become and remain a part of that sub-group as opposed to the norms of the larger society.

Hence, factors such as peer conformity, group norms, beliefs and misconceptions all play a critical role in SCT.

The desire to be accepted in a peer group is likely to entice an adolescent to conform to group norms such as sustained marijuana use. For instance, peer conformity has influenced juvenile delinquent behaviours such as indulgence in petty crimes and drug use.^26^ This is so because when indulgence in delinquent acts such as drug use becomes an acceptable norm for a group, members are obliged to participate or be ostracised.

Moreover, beliefs and misconceptions towards illicit drug use amongst subcultural groups also promote the behaviour. Adolescents, through their peer associations, are often made to believe that marijuana use is not harmful but has many unverified social and health benefits.^26^ For example, most marijuana users in the United States believe that the drug is non-psychoactive.^27^ Should adolescents in economically marginalised settings of South Africa, where illicit marijuana is cultivated and traded, hold onto some of these misconceptions, they could lead to sustained illicit marijuana use.

We therefore sought to empirically ascertain the contextual factors of sustained adolescent marijuana use in the Ingquza Hill Local Municipality of South Africa, using the tenets of DOT and SCT. This is to add to the body of knowledge on theoretical explanations for adolescent marijuana use to better inform policy on the prevention of adolescent marijuana use in similar contexts where the plant is illegally grown and consumed.

## Methodology

### Study setting

The study was conducted in two communities in the Ingquza Hill Local Municipality of the Eastern Cape Province of South Africa. The municipality has an estimated population of 278 481 people, with a population density of 234 people per square kilometre.^28^ The communities were purposively chosen for the study because they are known for engaging in illegal marijuana cultivation and trade.^12,13^ Consequently, adolescent marijuana use is perceived to be rife in these communities,^29,30^ and therefore, they were viewed as appropriate grounds for data collection in answering the research question.

### Study design

Using the focus group discussion data collection approach, the exploratory qualitative research design was used to explore the contextual influences of sustained adolescent marijuana use in two selected communities in the Ingquza Hill Local Municipality of South Africa. According to Wyk,^31^ the exploratory research approach focusses on understanding issues with high levels of uncertainty and ignorance, such as why some adolescents in illicit marijuana-growing communities use marijuana, whilst others do not. The focus group data collection approach was also used as it allowed the researchers to observe areas of strong emphasis, especially in aspects that discussants were most passionate about.^32^ Focus group members were people who knew each other and were active marijuana users at the time of conducting the study.

### Participants

Thirty-seven participants, all male participants aged between 14 and 19 years who were residents of any of the two communities and currently smoked or used marijuana in any form, were involved in the study. Thirteen were aged between 14 and 16 years, whilst 24 were aged between 17 and 19 years. In total, four focus groups were formed. Each community had two focus groups. The first community (Community 1) had 19 participants, 9 in the first focus group and 10 in the second group, whilst the second community (Community 2) had 18 participants.

Hence, each group had nine members. With reference to education, 31 participants had been to secondary school, whilst the remaining six participants were primary school dropouts.

All participants were not formally employed and were dependent on their parents or guardians for survival. Two focus groups per community, resulting in four groups overall, were formed, ranging from 6 to 12 participants per focus group. Recruitment and participation in the study took into account societal stigmatisation associated with marijuana use.^33^ As a result, the number of qualified participants obtained from each community could only form two groups per community.

### Participant recruitment

A two-staged non-probability sampling procedure was followed to recruit participants. Firstly, purposive sampling was used to select the two communities from a list of communities under the Ingquza Hill Local Municipality.

The variable of interest was communities where illicit marijuana activities were rife and where adolescent marijuana use was bound to prevail. To conceal participants’ identity from their legal guardians, verbal permission was sought from community members (including guardians) for participants who were below 18 years through community tribal courts.

Key informants were then identified in each community after the researcher had lived in each community for up to a month. Once a key informant was identified and recruited, the snowball sampling technique was used to recruit the rest of the participants. This was performed by referring researchers to a known and willing marijuana user. Thus, key informants, together with initially recruited participants, helped to identify subsequent participants who met the inclusion criteria.

This sampling method was adopted as the appropriate method to penetrate the circles of marijuana users in communities where there is some form of stigma associated with the behaviour under investigation. Participants were recruited and interviewed in a secluded location, out of sight of their legal guardians, community members and members of other focus groups.

Assigning participants to a group was performed based on the association a participant had with other participants. Thus, participants who belonged to a social network were grouped together. This offered some sense of security and protection to participants and enabled them to open up during interviews. However, the comparability of the groups was ensured by recruiting only active marijuana smokers or users who shared similar socio-demographic characteristics.

### Data collection tool

Data were collected using a pre-tested semi-structured interview guide. The guide contained questions on two broad areas: socio-demographic characteristics of participants and their motivations for marijuana use. The guide was developed in the IsiXhosa language, translated into English and translated back into the IsiXhosa language. This was to ensure that the questions did not lose their meaning during translation.

#### Data collection procedure and ethical considerations

We ensured that the study’s ethical procedures did not go beyond institutional requirements for ethical clearance by adhering to the principles of ethical reflexivity.^34^ Firstly, we met the institutional requirements for ethical clearance by obtaining an ethical clearance certificate for the study from the Higher Degrees Ethics Committee of Walter Sisulu University, with protocol number 047/2013. There were also considerations for traditional and cultural requirements in the two study sites before data collection begun. Also, we presented the project to the various community leaders in the selected communities by following the necessary entry processes. Being mindful of the sensitive nature of the study, we had to gain the trust of community members for them to open up to interviews and ensure our own security and welfare. After meeting with various community leaders to introduce ourselves and the project to them, we proceeded to hold community briefings with community members to inform them of our presence in their communities and why we were there. Although they were initially apprehensive, we were eventually accepted into the communities to conduct the study. To obtain credible results, we stayed in each community for up to a month before data collection began. This was to help us build social relationships with community members who would lead to working relationships.^34^ This was achieved by visiting social event sites in the communities such as eateries and clubs, where conversations were forged with community members. We also offered help to community members in the form of transportation.

A trusted key informant then helped with the selection of initial participants (marijuana smokers).

After recruiting the first participants, we were led to other marijuana smokers in their inner circles.

Verbal consent was also sought from parents and guardians of participants below 18 years who partook in the study during community forum discussions. This was, however, on the condition that participants voluntarily agreed to partake in the study, and their rights were spelt out to them.

We then sought participants’ consent by informing them of their right of refusal to participate in the study, the benefits and risks associated with the study and how their privacy and anonymity would be protected. Participants who voluntarily agreed to participate in the study either signed or thumb printed the informed consent or child assent forms. Alphabets were used to represent the identities of participants, whilst numbers (1 and 2) were used to denote their respective communities for the purpose of anonymity.

Focus groups were then formed per each clique of marijuana smokers who had been recruited. Two focus groups per community were formed. Two trained research assistants, who were postgraduate students at the Faculty of Health Sciences of the Walter Sisulu University, fluent in English and IsiXhosa languages, collected the data in March 2016 under the principal investigator’s supervision. The assistants were trained on the data collection instrument and on the process of conducting focus group interviews by E., M.J.N and M.D. Each group members were assigned alphabets, which were used to identify them in relation to their bio-demographic characteristics, communities, focus groups and responses.

To ensure methodological reflexivity, we ensured that the interview process was dynamic and not static. For instance, we started with a structured interview guide. However, upon the first interview, we identified that the approach will make the interviewer’s voice overly dominant over the interviewee’s voice and salient contextual information. We then resorted to a semi-structured interview guide to create room for the interviewer’s voice to be proportionally represented.^35^ There was also a dialogue between the researchers to clarify and understand the participant’ questions and responses. This was particularly necessary as the principal investigator (PI) did not understand the IsiXhosa language in which the interviews were conducted but relied on the interviewers to understand discussions to ensure that the interviewers were on track in terms of the objectives of the study. We also kept a reflective journal for introspection so that mistakes that were made in previous interviews or issues omitted were clarified in subsequent interviews. Each group discussion lasted for about an hour and was recorded with an Olympus voice recorder, with the participants’ permission.

### Data analysis

The thematic content analytical technique was used to analyse the data. The voice recordings were translated and transcribed from IsiXhosa into English by a qualified language translator from the Eastern Cape, Department of Basic Education, South Africa. During the translation process, there was a constant dialogue between the researchers and the interpreter to ensure that the interviewee’s exact words were used for interpretation to further ensure methodological reflexivity. Content analysis is an analytical process that applies intuitive and interpretive approaches to systematically summarise textual data.^36^ Content analysis was preferred because it enables systematic coding of data by organising the information into categories to discover patterns undetectable by merely listening to the tapes or reading the transcripts.^37^ The data sets were first labelled to keep track of their source, the communities and focus groups they emanated from. The transcripts were then thoroughly read to gain a general sense of the information. Each transcript was put into segments and codes, which were descriptive in nature in terms of the subject matter of the transcript. Coding was performed by writing the applicable codes in the margins of transcripts. Coding was performed by E.M. and M.D. independently. The first and second authors then reviewed discrepancies in the double-coded interviews and revised discrepancies through consensus using the most expressive words for each set of codes. We then grouped related topics under various themes, based on existing thematic areas reported in the literature and those that emerged from the data analysis process and were unique to this study.^38^ Themes were identified by the first and second authors (E.M. and M.D.) and reviewed by all authors. When further coding was not possible, data saturation was deemed to have been reached.^39^ Nine themes (motivations) emerged from the analysis. They are availability and affordability of marijuana, peer conformity, pleasure, idleness, addiction, manipulation of appetite as a means of dealing with personal problems, health reasons and higher cognitive function. The results, with their supporting excerpts, are presented under these themes.

## Trustworthiness of the study’s findings

According to Guba,^40^ four key questions need to be addressed in terms of trustworthiness. These include truth value (also credibility), applicability (transferability), consistency (dependability) and neutrality (confirmability).

The credibility of the study’s findings was achieved by staying long enough in our study communities to gain participants’ trust and opening them up for interviews, and to engender credible responses. This also allowed for on-field member checking by going back to participants with the transcribed data to ensure that what was written was exactly what they had said or meant during the interview process as the research process was iterative. We also resorted to peer debriefings where experienced colleagues in qualitative research were consulted, and their inputs were then incorporated into the research process to make the findings more credible.

Transferability, on the other hand, was ensured through a detailed description of our study setting, procedure, context and data to make it possible for our findings to be compared to those from contexts with similar characteristics, although that is not the aim of the naturalistic inquiry.^40^

Moreover, the dependability of the findings was ensured through stepwise replication of study findings. The data sets were analysed by two authors (E.M. and M.J.) independent of each other, after which meetings were arranged to discuss and merge study findings. Before the formal meetings, consistent communication took place between the researchers to cross check developing insights and make decisions about the appropriate steps. The research team also kept an audit trail of all activities of the study, from community entry to data analysis. These documents were later given to an external auditor from another university to analyse how data were collected, analysed and interpreted. Lastly, we ensured confirmability by being transparent with our study participants by practising reflexivity. Thus, we revealed to our participants why we formulated and presented our questions and study findings the way we did. We also arranged for a confirmability audit that certified that data existed in support of every interpretation and that the interpretations were made in a manner consistent with available data.

### Ethical considerations

Ethical clearance for the study was granted by the Higher Degrees Ethics Committee of Walter Sisulu University, with protocol number 047/2013. Permission, in the form of verbal consent, because of the sensitivity of the topic and the need to conceal the identities of communities and participants, was then sought from all relevant stakeholders, and thus, community elders through community entry and community members through community forums after obtaining ethical clearance. Verbal consent was also sought from parents and guardians of participants who were below 18 years who partook in the study during the community forum discussions. This was, however, on the condition that participants voluntarily agreed to partake in the study and their rights spelt out to them. Participation was therefore voluntary and participants’ right to withdraw from the study was made known to them and respected. Individual participants were assured of anonymity as alphabets were used to represent their identities, whilst numbers (1 and 2) were used to denote their respective communities. Participants derived no direct benefits from the study and were neither exposed to risks in the course of the study

## Results

Nine reasons were given by participants about why they continue to use marijuana, which have been grouped into differential opportunity and sub-cultural factors. Differential opportunity factors include availability and affordability of marijuana, idleness and as a means of dealing with personal problems. Subcultural factors include peer conformity, the pleasure derived from smoking marijuana, manipulation of appetite, health reasons, higher cognitive function and addiction.

### Motivations for sustained marijuana use

#### Differential opportunity influences of sustained marijuana use

**Availability and affordability of marijuana:** Participants indicated that the availability of marijuana in their communities made it affordable, requiring little or no amount to have access to it, enabling them to smoke marijuana at will. They also had developed the skills of using marijuana early in life because of its availability in their environs, thus becoming expert users and their drug of choice. Some discussants explained:

‘Buying it [*marijuana*] is very easy because it is easily accessible and cheap. When you have R2.00, you will come across someone, and you will be able to get it. It is very cheap compared to other stuff [*drugs*]. Sometimes even if I don’t have money, I am still able to get my nkantinin [*marijuana*] to smoke. I can get it [*marijuana*] on credit and pay later. Sometimes I pretend like I don’t owe you [*the seller*], and that’s it. I don’t pay for it any more.’ (Participant 33, male, secondary school graduate, 19 years old)

‘I prefer dagga [*marijuana*] because it is the drug I have been using all this while, so I know everything about it; how it works in your body and the benefits you [*one*] get from it, so I don’t see myself using any other drug. In fact, it will be very difficult to stop smoking it.’ (Participant 11, male, student, 18 years old)

Therefore, the availability of the drug in their contexts makes it accessible and affordable for adolescents, leading to its early and sustained usage amongst discussants.

#### Idleness (lack of economic opportunities)

Because of the lack of economic opportunities in the communities, participants are idle, meaning that they have much free time. Therefore, smoking marijuana has become one way of killing boredom. Two participants retorted:

‘The problem is that you wake up the whole day, and there is nothing here to do. If the government had provided us with jobs, at least, we will be occupied somehow and not smoke that much.’ (Participant 26, male, secondary school graduate, 18 years old)

‘We [*the youth*] are not working, so we have time for smoking. We are not schooling too, so we wake up not knowing what to do, and the only thing that comes to your mind is to go to your friends and have a smoke [*of marijuana*].’ (Participant 31, male, secondary school graduate, 19 years old)

The lack of employment opportunities in the area, coupled with a lack of mobility on the part of the discussants, made them to have enough unwanted and unproductive free time to channel into habitual marijuana smoking.

#### Means of dealing with personal problems

The participants revealed that they used marijuana to forget about their problems, as that was the only way they knew to handle the numerous socio-economic issues that bothered them:

‘My brother, I won’t lie to you, “nkanthini” [*marijuana*] can make you forget about many of your problems. When I have a quarrel at home with my parents and don’t want to do something stupid, I just go out to smoke.’ (Participant 15, male, student, 17 years old)

‘Marijuana takes your problems away. Sometimes you are stressed, and you need to calm down with dagga [*marijuana*], so it is a lifesaver. As you can see, there is nothing going on here for us [*in terms of economic opportunities*], so dagga is our companion and comforter.’ (Participant 29, male, secondary school dropout, 18 years old)

The economic and social despondency of discussants in their various contexts leads them to find solace in marijuana smoking to deal with their problems as they were hopeless under their precarious economic conditions.

### Subcultural influences of sustained marijuana use

#### Peer conformity

Being in the company of peer, marijuana smokers served as an important subcultural influence of marijuana use amongst discussants. In the form of a group norm, discussants felt obliged to smoke marijuana to conform. They explained:

‘You know, when it is time to go and have a smoke, you cannot stay behind because you are part of the [*peer*] group; it [*marijuana smoking*] is some kind of ritual we perform as amajita [*young men*].’ (Participant 4, male, student, 17 years old)

‘I will say it is because I have so many friends that smoke it [*marijuana*]. Even on days that I am not in the mood [*to smoke*], a friend will just appear from nowhere with some [*marijuana*], and you immediately feel for it because if you don’t do it [*smoke*], it may seem like you are better than them. So, once I have friends here [*in this community*] who are smoking, only God can help me [*to quit smoking*].’ (Participant 20, male, student, 18 years old)

It is obvious from the above quotes that participants’ peers exert enormous pressure on them to smoke marijuana to conform to group norms.

#### The pleasure of marijuana smoking (Euphoria)

We found that some adolescents continue to smoke marijuana because of the good feeling they obtain from smoking it. They therefore see non-marijuana smokers as lacking something precious in life as they miss out on this euphoria. Hence, marijuana smoking is a subcultural activity as only those in that culture can enjoy such euphoria as words cannot explain it. They recounted:

‘I can’t explain it [*the feel ing one gets from marijuana smoking*]; only my friends understand it [*the feeling*]. You get such a great feeling that you forget about everything in the world. It is amazing.’ (Participant 7, male, student, 19 years old)

‘I smoke marijuana because I get a good feeling from it. Because of marijuana, though I don’t have a girlfriend, I don’t feel it. All I need is to get my nkantini [*marijuana*], and I am good to go, no stress.’ (Participant 10, male, student, 17 years old)

Marijuana smoking brings a certain ecstasy to smokers, a feeling that is indescribable to ordinary people who do not smoke marijuana.

#### Manipulation of appetite

Discussants believed that marijuana served as both an enhancer and suppressor of appetite. Whilst some participants continued to smoke marijuana to have a great appetite for food, others do so to suppress hunger when they were in situations where food was not available:

‘As for me, it helps me to eat well. Once I have smoked my roll of dagga [*marijuana*], even if the food tastes horrible, I don’t care; I will finish it.’ (Participant 7, male secondary school dropout, 16 years old)

‘It [*marijuana*] makes me stay longer with the cattle in the veld [*grazing field*] without getting hungry. You will just be feeling thirsty, but then there is a river you can go and drink from.’ (Participant 22, male, student, 14 years old)

Deducing from the responses, discussants have varied beliefs about the role marijuana plays in appetite regulation. Whilst to some, it serves as an enhancer of appetite, to others, it suppresses appetite.

#### Health reasons

Some discussants cited health reasons for their continual marijuana use. According to them, there are certain ailments they suffer from, such as cough, tuberculosis and headaches, which are treatable by marijuana. Hence, they smoke it on the advice of their elders who are experienced in marijuana smoking to relieve them of their ailments. Some discussants explained:

‘I used to have this terrible cough every winter, so the elders used to say it [*marijuana*] helps with the cough as well as tuberculosis and shortness of breath, so smoke a little. Since I started smoking marijuana, I do not suffer from that terrible cough again.’ (Participant 14, male, secondary school graduate, and 19 years)

‘This thing [*marijuana*] is a medicine, my brother. There are a lot of health benefits from it, like the treatment of severe headache and pain, but sometimes you need someone who is experienced in this thing [*medicinal uses of marijuana*] to be able to tell you how to treat yourself with marijuana.’ (Participant 26, male, secondary school graduate, 18 years)

Discussants thus believe in the concept of medical marijuana, albeit it is based on the advice of experienced marijuana smokers, making it a subcultural belief.

#### Higher cognitive function

According to the discussants, marijuana use was associated with higher cognitive function. They believed that marijuana helps to sharpen one’s brain, and thus, improves academic performance. They explained:

‘I am a student, there is this subject called Mathematics. Once I smoke dagga [*marijuana*], I solve it easily. It [*marijuana*] helps me to deal better with numbers.’ (Participant 12, male, student, 15 years old)

‘One thing with marijuana is that if you are clever, it makes you cleverer. You are able to think faster and solve problems. I know many boys who became more brilliant at school when they started smoking marijuana.’ (Participant 31, male, secondary school graduate, 19 years old)

Discussants believed that to perform better in challenging subjects like mathematics, one needs to smoke marijuana, influencing their decision to keep using the drug.

#### Addiction

Because of the early and sustained usage of marijuana resulting in people’s dependency on it to deal with idleness and personal problems, discussants have become addicted to the drug, making it difficult for them to quit its usage. They explained:

‘… It [*marijuana*] is available, we end up stealing and smoking it [*marijuana*], and now we are addicted. I am not sure I can stop [*smoking*]. My blood is full of marijuana now.’ (Participant 18, male, student, 19 years old)

‘We have become used to this thing [*marijuana smoking*], so how can we stop [*smoking*]? Tell me, my brother. For some of us, unless they remove us from this place [*community*] and take us [*marijuana smokers*] to a government facility [*rehabilitation centre*]. Maybe that will help [*to quit marijuana smoking*]. You wake up every day, and there is nothing for you to do, so as a man, you have to smoke [*marijuana*] to keep yourself busy.’ (Participant 33, male, secondary school graduate, 19 years old)

The early and sustained usage of marijuana by discussants has therefore led to their addiction, making it difficult for them to quit its usage.

## Discussion

In this study, we have investigated motivations for sustained marijuana use amongst adolescents from two South African marijuana-growing communities, using the DOT and SCT constructs.

Differential opportunity theory postulates that aside from the set of skills that one possesses, other routes of marginalisation and disenfranchisement of communities such as unemployment, lack of economic opportunities and mobility lead to sustaining illicit behaviours, such as marijuana use, especially in environments where illicit cultivation and trading of the plant abound.^21^ Subcultural theory, on the other hand, suggests that deviance results from individuals conforming to the values and norms of a social group to which they belong.^25^ Analysis of the data revealed nine motives, which were grouped under the DOT (availability and affordability, idleness and means of dealing with personal problems) and SCT (peer conformity, the pleasure derived from marijuana smoking, manipulation of appetite, health reasons, higher cognitive function and addiction) that sustain marijuana use amongst discussants.

Differential opportunity theory factors such as availability and affordability of marijuana were strong drivers of sustained adolescent marijuana use. The participants reported that marijuana is easily available and affordable in their communities. The average unit cost of cannabis in South Africa is about $ 8.5 and comparatively cheaper, making it affordable for adolescents^41^ and could therefore influence sustained marijuana use amongst adolescents. Affordability and availability of marijuana have been found to lead to sustained marijuana use in the Americas and Asia,^42,43^ buttressing our finding.

Moreover, in some communities, marijuana is used in the presence of children and adolescents, influencing them to adopt its usage.^44^ Adult marijuana use in homes and communities promotes continued adolescent marijuana use as they may steal from adult users.^45,46,47^ Marijuana availability and accessibility in vulnerable communities therefore need to be addressed to curtail sustained adolescent marijuana use in such environments.

We also found idleness as another DOT motivation for the constant use of marijuana amongst discussants. Unemployed adolescents have much spare time. As a result, they spend a good part of the day engaging in the use of marijuana. For others, to escape boredom associated with idleness, they find a way out through the instant gratification that marijuana smoking provides.^48^

This is because it is their only way of having fun. Similar findings have been reported elsewhere.^49,50^ The lack of employment opportunities in the communities plays an important role in sustained adolescent marijuana use. Therefore, authorities should strive to engage unemployed adolescents in economic ventures that would make them less idle, so they have less time for marijuana use.

Furthermore, sustained adolescent marijuana use was found to be another DOT means of dealing with personal problems. Discussants indicated that problems such as family pressure resulting from economic dependency, which leads to stress and anxiety, make them resort to marijuana use for solace. Similar findings have been reported in Canada.^51^ Adolescents have often hinted that they could take their minds off their personal problems once they smoke marijuana. Thus, for adolescents in deprived settings faced with economic and personal problems, anger or depression, getting high on marijuana becomes their choice for finding relief.^51,52^ This finding brings to the fore the lack of viable economic ventures and professional counselling services for the youth in deprived communities where illicit drug activities are rife. As adolescents from such backgrounds have no professional means of dealing with their problems or active economic engagement to deal with their problems, they turn to marijuana use. Efforts therefore need to be made to improve socio-economic conditions in deprived marijuana-growing settings of South Africa whilst at the same time providing counselling sessions on coping mechanisms to such adolescents.

Meanwhile, some SCT factors were also found to influence adolescent marijuana use in marijuana-growing contexts of South Africa. For instance, peer conformity was a strong SCT influence that promoted sustained marijuana use amongst discussants. It was found that some discussants wanted to fit in with their peers.^44^ The desire to keep being recognised as a true member of one’s social circle pushes adolescents to continue using marijuana.

In the majority of these circles of friends, those who use marijuana are considered very popular. Thus, in their quest to attain such levels of popularity, some adolescents also tend to use marijuana. Studies have reported that practices regarding marijuana use are usually introduced by categories of individuals, including one’s peers.^45,49^

Sustained marijuana utilisation in the current study is largely influenced by normative peer group culture as adolescents who look up to popular members amongst their peers continue to take marijuana in order to be accorded the same level of recognition.^53,54,55^

We also found that the pleasure associated with the use of marijuana was another SCT factor that reinforced marijuana use amongst discussants. This kind of pleasure, discussants explained, could only be understood by people who are marijuana smokers. The use of marijuana for pleasure has been documented in the literature as marijuana has been noted to interact with certain neurotransmitters that stimulate pleasure.^56^ Hence, persistent use of marijuana as a result of its ability to give pleasure leading to dependency has been corroborated by research.^57,58,59^ The instant gratification experienced by marijuana users gradually makes it difficult for adolescents to discontinue its use. Thus, the pleasure derived from the use of marijuana negatively reinforces its use amongst adolescents. Marijuana-driven pleasure may be a subculture because it has been reported that adolescents who depend on the drug for pleasure may be suffering from dependency syndrome,^60^ a condition not found in the general population. This syndrome therefore needs to be interrupted through rehabilitation in order to curtail sustained marijuana use.

Sustained adolescent marijuana use was also found to be motivated by the desire to enhance or suppress one’s appetite, a belief of the marijuana-smoking subculture. Whilst hormonal and pharmacological interactions of marijuana regarding appetite have been documented,^61,62,63^ it has not been recommended as the appropriate means of manipulating appetite. Our study found that adolescents who engaged in menial jobs at which they needed to spend long hours without food used marijuana to help them to suppress their appetite. On the contrary, some others use it to boost their appetite. Thus, the evidences for the effect of marijuana on hunger and appetite are mixed and need to be clarified amongst adolescents in marijuana-growing settings.^63,64^

Moreover, some adolescents used marijuana because of the perceived health benefits that they associate with marijuana use. The use of marijuana for health reasons has been scientifically documented.^61,62^ However, the medical prescription and use of medical marijuana are only based on rigorous and extensive research evidence.

One has to be medically certified or recommended to use marijuana for medical purposes. Nevertheless, in rural South African contexts where herbal medicinal use is a common practice and marijuana is grown, the plant is often recommended by those who use it to treat minor ailments such as cough.^13^ This creates the impression amongst adolescents that marijuana use is not detrimental to their health but contains medicinal properties leading to poor health-seeking behaviour.

Moreover, we found that discussants continue marijuana use because of its supposed ability to give them a higher cognitive function. Amongst discussants who were high students, especially, there was the notion that marijuana use can boost one’s memory and increase their ability to answer questions in class and during an examination.

This finding was consistent with the literature as adolescents reported that marijuana use helps them to focus or concentrate on their academic work.^49,65^ Whilst this misconception is unfounded, the literature evinces that it is common for substance users to attach some benefits to the kind of substance they persistently use to justify their actions even though such justifications may not be scientifically proven.^66^ Scientific evidence, on the contrary, suggests that marijuana actually impairs cognitive function.^67^ Hence, if not addressed, this perception could reinforce marijuana usage over time amongst schoolchildren leading to addiction.^68^

Lastly, addiction was another SCT motivation for sustained marijuana use amongst discussants.

Drug addiction is defined as a ‘chronically relapsing disorder’ marked by compulsive drug seeking and intake, loss of control in limiting intake and the emergence of a negative emotional state when access to a drug is prevented.^56^ Addiction to substance use has been largely studied in the scientific literature.^56,69,70^ Over time, adolescents who use marijuana become highly dependent on it, which makes it difficult for them to live without it.^68^ Typically, they cannot do without marijuana for a day as a result of the skills and joy they have developed in the act. There is therefore the need to pay attention to contextual factors that promote marijuana use leading to addiction amongst adolescents in illicit marijuana-growing contexts of South Africa.

## Conclusion

The study identified multifaceted motives for sustained adolescent marijuana use, which were grouped under DOT and SCT. The findings have implications for adolescent marijuana use prevention in the Ingquza Hill Local Municipality and similar contexts. As marijuana has been identified to be a gateway drug to the use of other illicit drugs, its sustained usage amongst adolescents poses a health challenge to both the user, their community and the country’s healthcare system at large. Hence, there is the need to intensify adolescent marijuana use prevention campaigns in illicit marijuana-growing contexts of South Africa, focussing on the differential opportunities and subcultural inclinations that promote the behaviour in those contexts.
